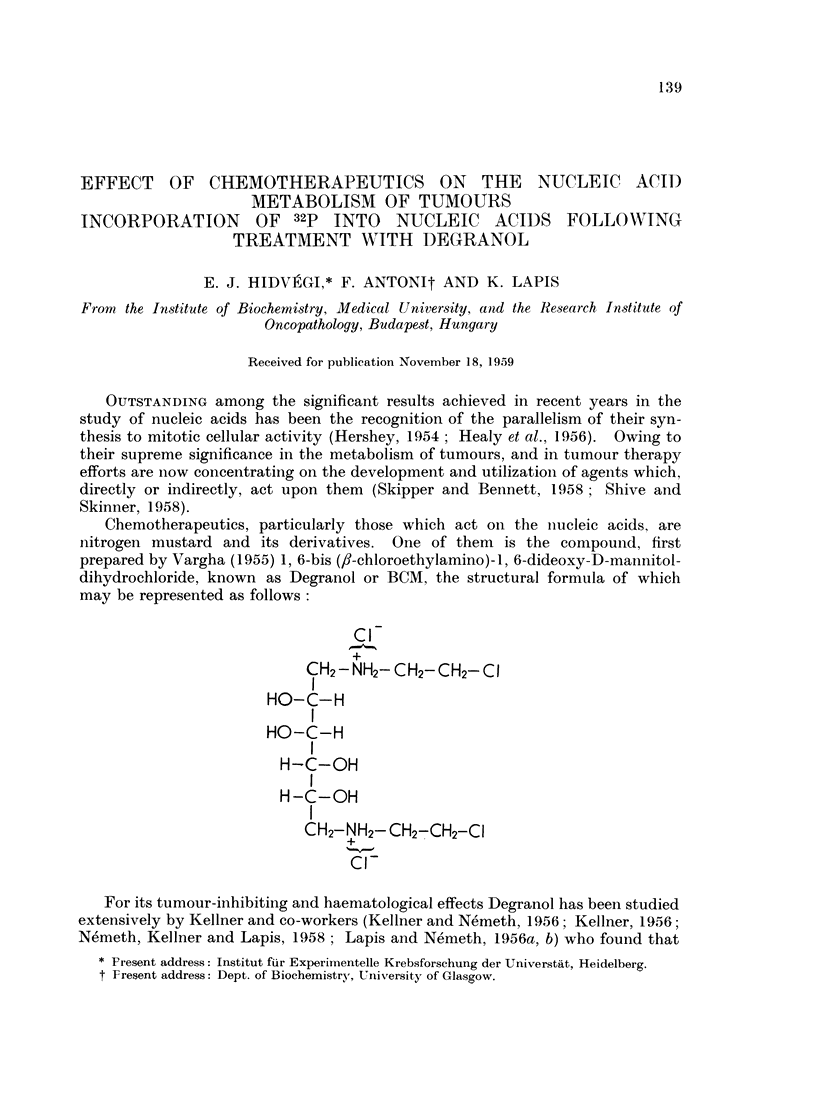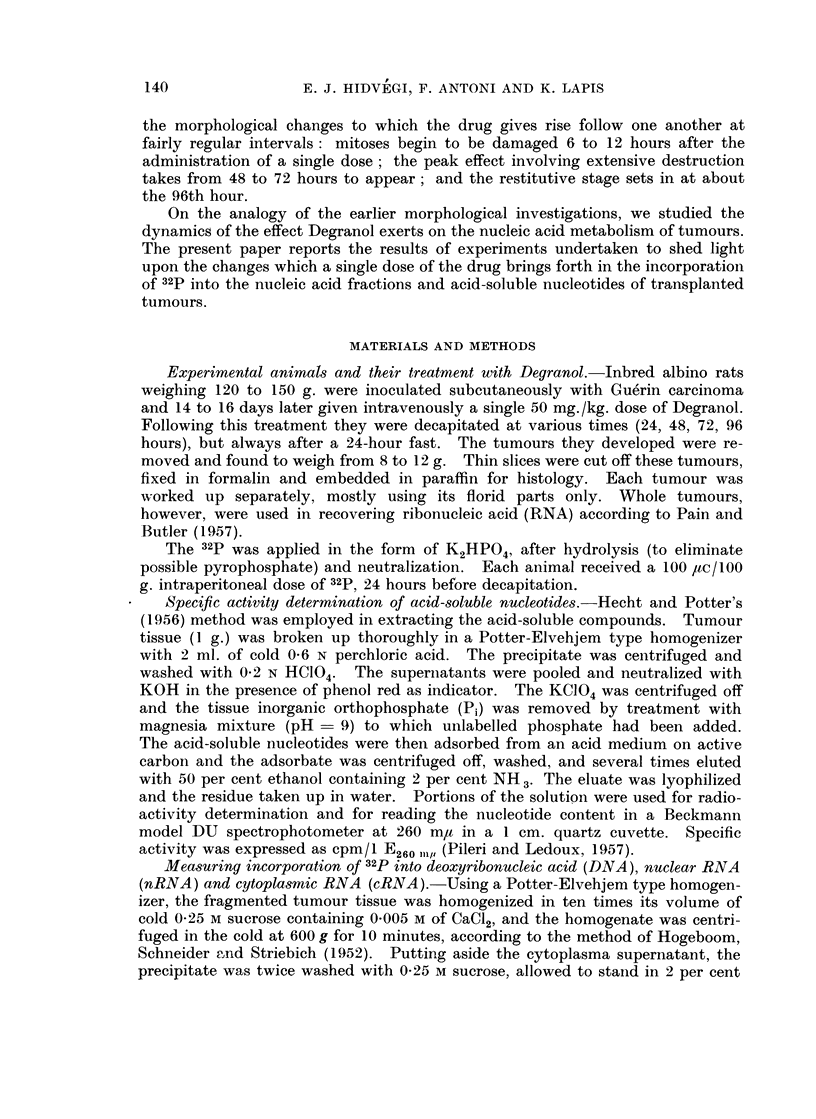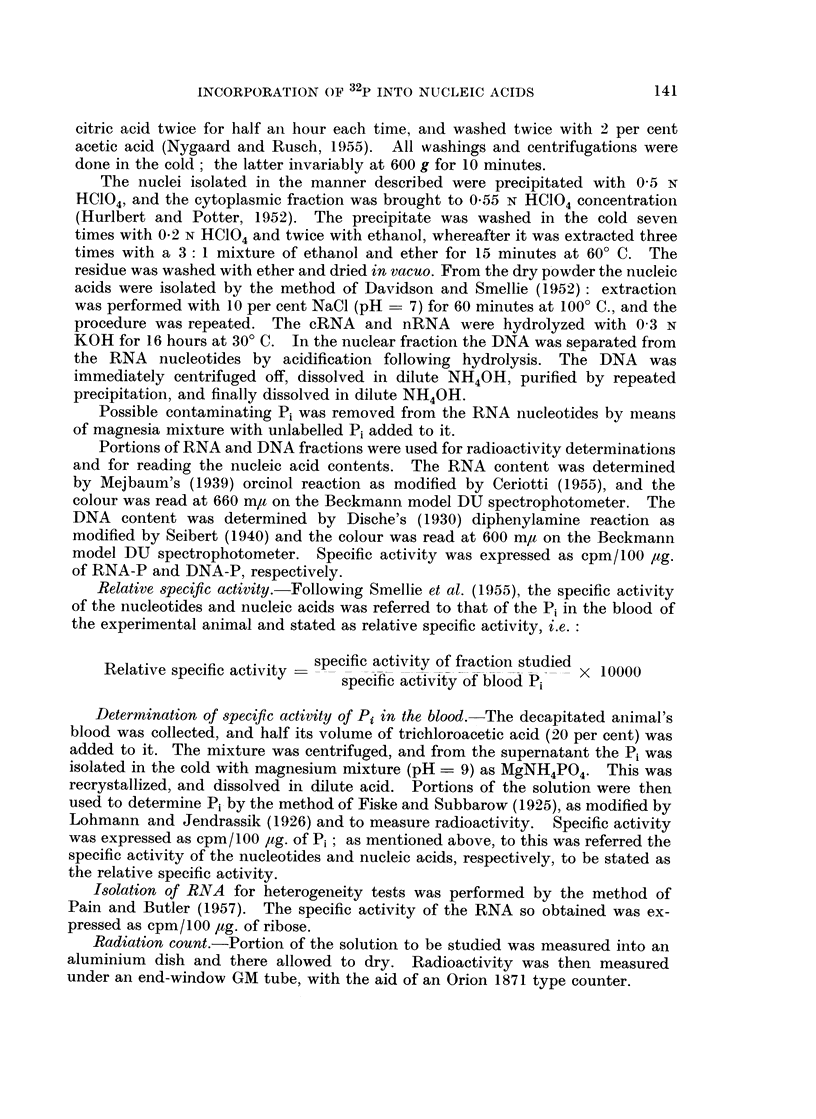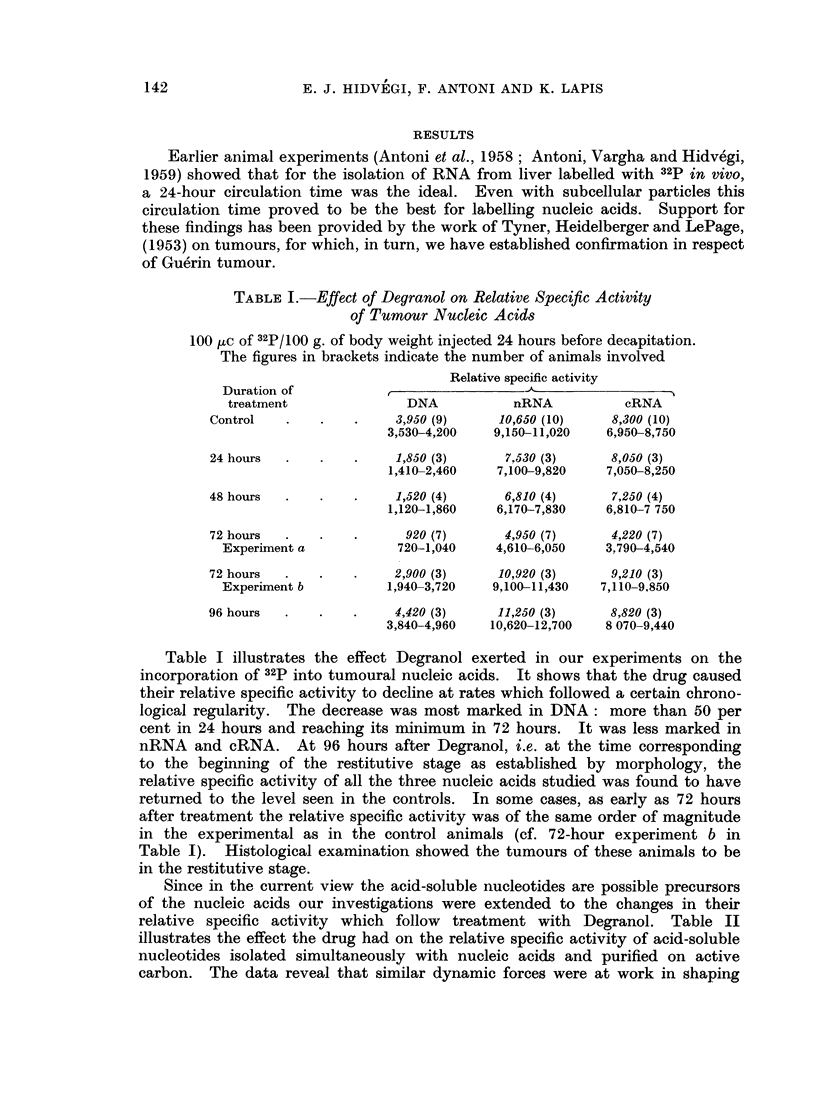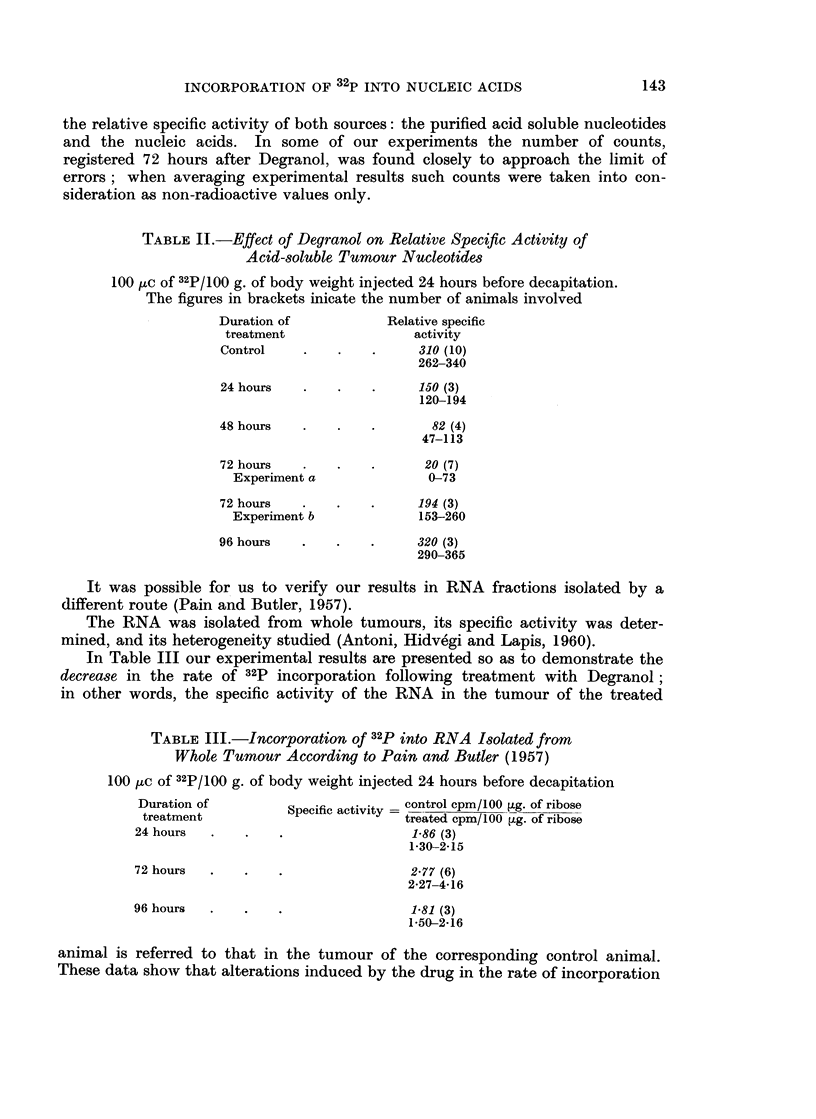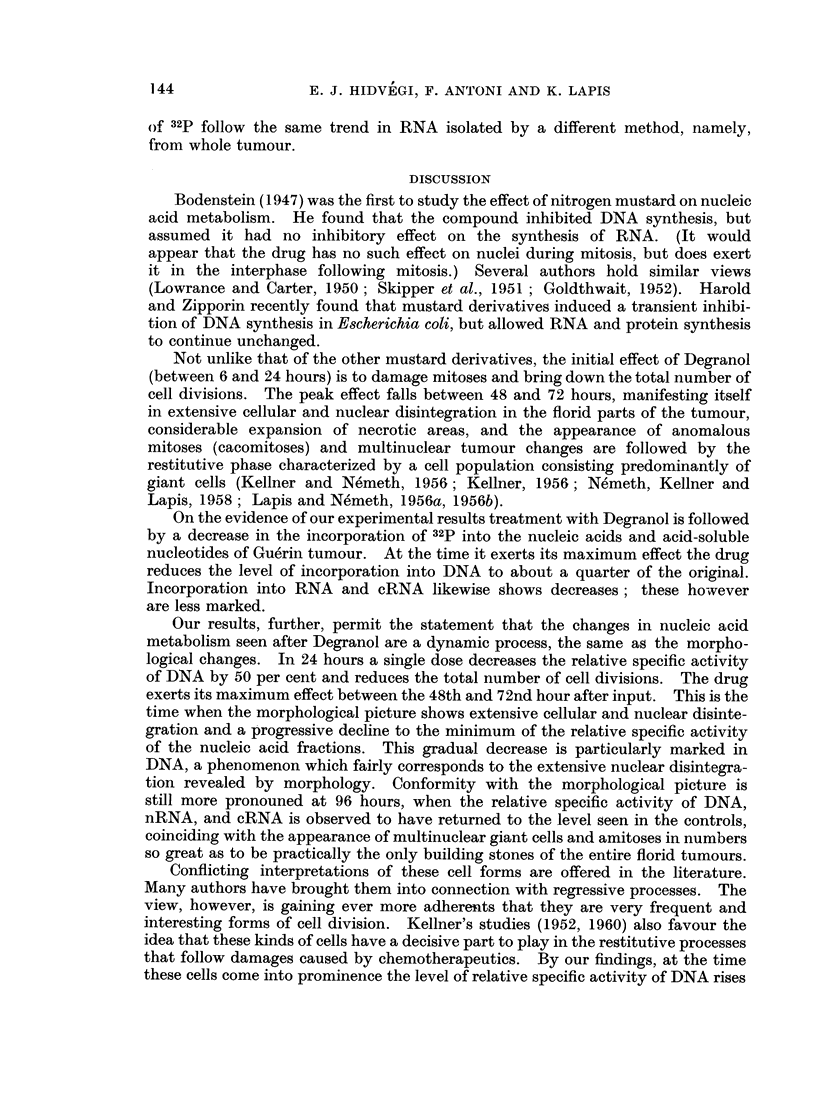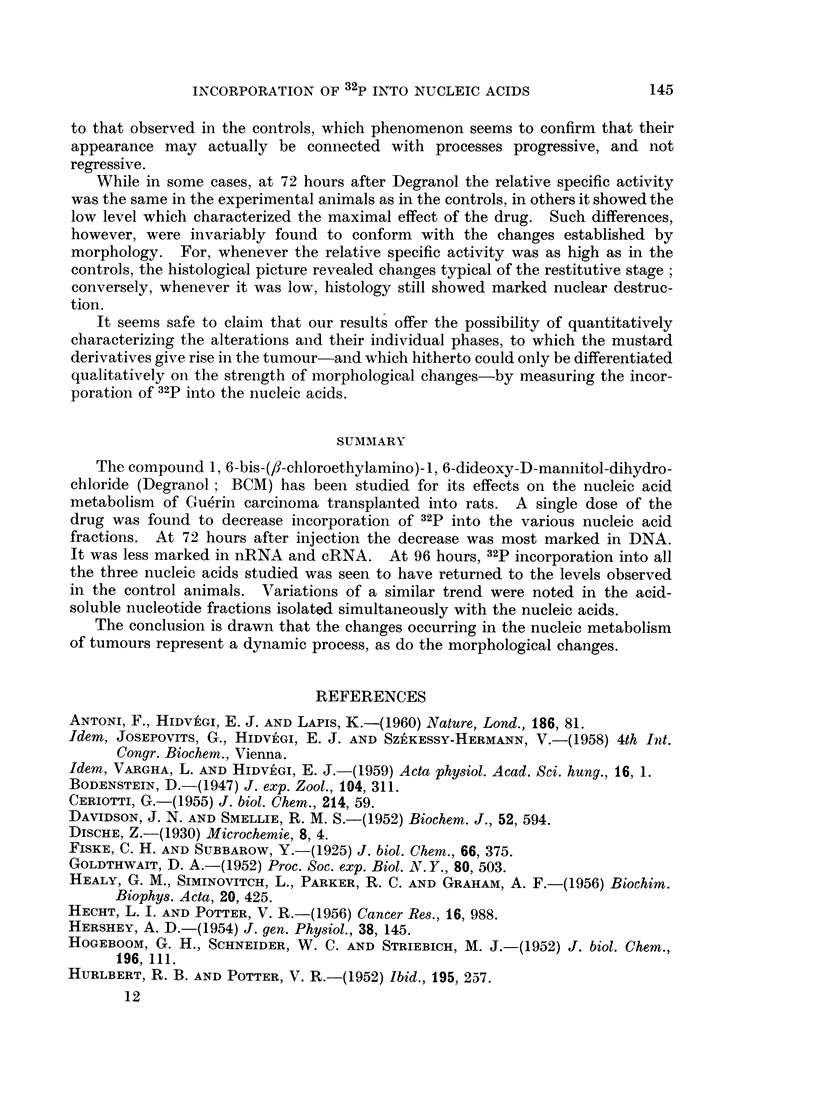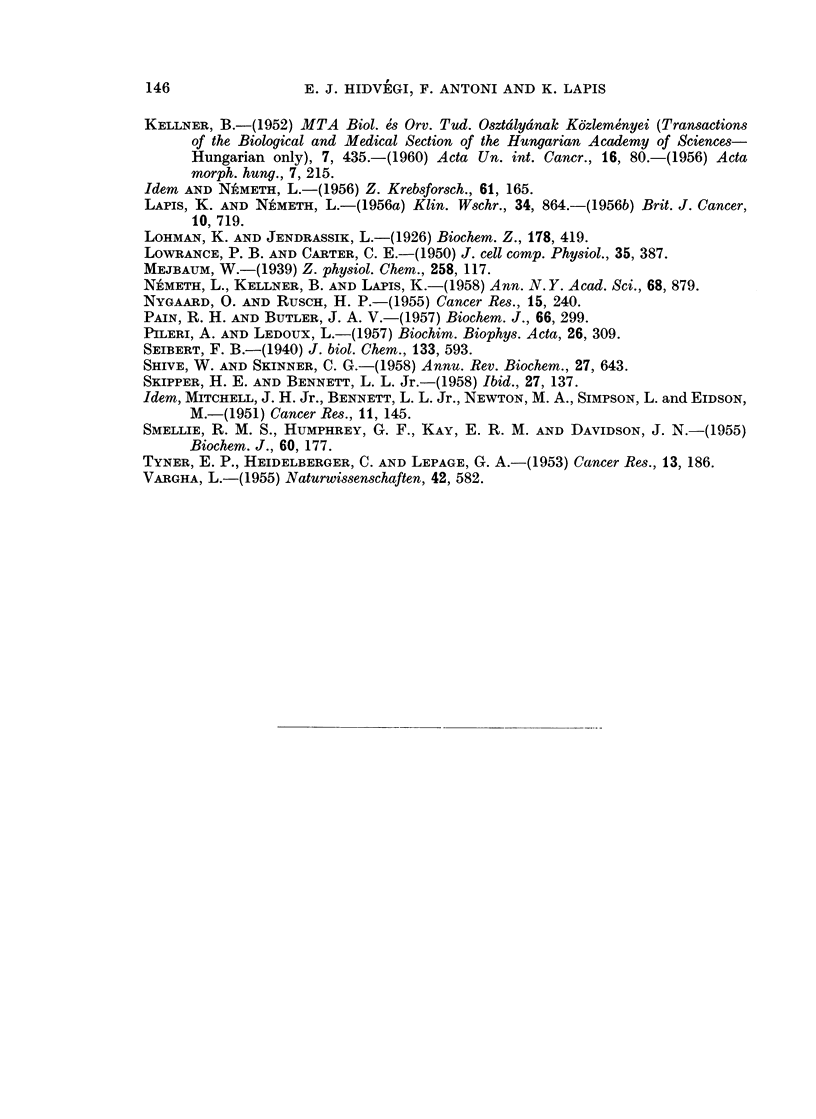# Effect of Chemotherapeutics on the Nucleic Acid Metabolism of Tumours. Incorporation of ^32^P into Nucleic Acids Following Treatment with Degranol

**DOI:** 10.1038/bjc.1960.16

**Published:** 1960-03

**Authors:** E. J. Hidvégi, F. Antoni, K. Lapis


### EFFECT OF CHEMOTHERAPEUTICS ON THE NUCLEIC ACID METABOLISM OF TUMOURS
### INCORPORATION OF $^{32}P$ INTO NUCLEIC ACIDS FOLLOWING TREATMENT WITH DEGRANOL

E. J. HIDVÉGI,* F. ANTONI† AND K. LAPIS

*From the Institute of Biochemistry, Medical University, and the Research Institute of Oncopathology, Budapest, Hungary*

Received for publication November 18, 1959

OUTSTANDING among the significant results achieved in recent years in the study of nucleic acids has been the recognition of the parallelism of their synthesis to mitotic cellular activity (Hershey, 1954 ; Healy *et al.*, 1956). Owing to their supreme significance in the metabolism of tumours, and in tumour therapy efforts are now concentrating on the development and utilization of agents which, directly or indirectly, act upon them (Skipper and Bennett, 1958 ; Shive and Skinner, 1958).

Chemotherapeutics, particularly those which act on the nucleic acids, are nitrogen mustard and its derivatives. One of them is the compound, first prepared by Vargha (1955) 1, 6-bis (β-chloroethylamino)-1, 6-dideoxy-D-mannitol-dihydrochloride, known as Degranol or BCM, the structural formula of which may be represented as follows :

For its tumour-inhibiting and haematological effects Degranol has been studied extensively by Kellner and co-workers (Kellner and Németh, 1956 ; Kellner, 1956 ; Németh, Kellner and Lapis, 1958 ; Lapis and Németh, 1956*a*, *b*) who found that

* Present address: Institut für Experimentelle Krebsforschung der Universtät, Heidelberg.
† Present address: Dept. of Biochemistry, University of Glasgow.

the morphological changes to which the drug gives rise follow one another at fairly regular intervals: mitoses begin to be damaged 6 to 12 hours after the administration of a single dose; the peak effect involving extensive destruction takes from 48 to 72 hours to appear; and the restitutive stage sets in at about the 96th hour.

On the analogy of the earlier morphological investigations, we studied the dynamics of the effect Degranol exerts on the nucleic acid metabolism of tumours. The present paper reports the results of experiments undertaken to shed light upon the changes which a single dose of the drug brings forth in the incorporation of $^{32}P$ into the nucleic acid fractions and acid-soluble nucleotides of transplanted tumours.

### MATERIALS AND METHODS

*Experimental animals and their treatment with Degranol.*—Inbred albino rats weighing 120 to 150 g. were inoculated subcutaneously with Guérin carcinoma and 14 to 16 days later given intravenously a single 50 mg./kg. dose of Degranol. Following this treatment they were decapitated at various times (24, 48, 72, 96 hours), but always after a 24-hour fast. The tumours they developed were removed and found to weigh from 8 to 12 g. Thin slices were cut off these tumours, fixed in formalin and embedded in paraffin for histology. Each tumour was worked up separately, mostly using its florid parts only. Whole tumours, however, were used in recovering ribonucleic acid (RNA) according to Pain and Butler (1957).

The $^{32}P$ was applied in the form of $K_2HPO_4$, after hydrolysis (to eliminate possible pyrophosphate) and neutralization. Each animal received a 100 $\mu$c/100 g. intraperitoneal dose of $^{32}P$, 24 hours before decapitation.

*Specific activity determination of acid-soluble nucleotides.*—Hecht and Potter's (1956) method was employed in extracting the acid-soluble compounds. Tumour tissue (1 g.) was broken up thoroughly in a Potter-Elvehjem type homogenizer with 2 ml. of cold 0·6 N perchloric acid. The precipitate was centrifuged and washed with 0·2 N $HClO_4$. The supernatants were pooled and neutralized with KOH in the presence of phenol red as indicator. The $KClO_4$ was centrifuged off and the tissue inorganic orthophosphate ($P_i$) was removed by treatment with magnesia mixture (pH = 9) to which unlabelled phosphate had been added. The acid-soluble nucleotides were then adsorbed from an acid medium on active carbon and the adsorbate was centrifuged off, washed, and several times eluted with 50 per cent ethanol containing 2 per cent $NH_3$. The eluate was lyophilized and the residue taken up in water. Portions of the solution were used for radioactivity determination and for reading the nucleotide content in a Beckmann model DU spectrophotometer at 260 m$\mu$ in a 1 cm. quartz cuvette. Specific activity was expressed as cpm/1 $E_{260\ m\mu}$ (Pileri and Ledoux, 1957).

*Measuring incorporation of $^{32}P$ into deoxyribonucleic acid (DNA), nuclear RNA (nRNA) and cytoplasmic RNA (cRNA).*—Using a Potter-Elvehjem type homogenizer, the fragmented tumour tissue was homogenized in ten times its volume of cold 0·25 M sucrose containing 0·005 M of $CaCl_2$, and the homogenate was centrifuged in the cold at 600 *g* for 10 minutes, according to the method of Hogeboom, Schneider and Striebich (1952). Putting aside the cytoplasma supernatant, the precipitate was twice washed with 0·25 M sucrose, allowed to stand in 2 per cent citric acid twice for half an hour each time, and washed twice with 2 per cent acetic acid (Nygaard and Rusch, 1955). All washings and centrifugations were done in the cold ; the latter invariably at 600 *g* for 10 minutes.

The nuclei isolated in the manner described were precipitated with 0·5 N $HClO_4$, and the cytoplasmic fraction was brought to 0·55 N $HClO_4$ concentration (Hurlbert and Potter, 1952). The precipitate was washed in the cold seven times with 0·2 N $HClO_4$ and twice with ethanol, whereafter it was extracted three times with a 3 : 1 mixture of ethanol and ether for 15 minutes at 60° C. The residue was washed with ether and dried *in vacuo*. From the dry powder the nucleic acids were isolated by the method of Davidson and Smellie (1952) : extraction was performed with 10 per cent NaCl (pH = 7) for 60 minutes at 100° C., and the procedure was repeated. The cRNA and nRNA were hydrolyzed with 0·3 N KOH for 16 hours at 30° C. In the nuclear fraction the DNA was separated from the RNA nucleotides by acidification following hydrolysis. The DNA was immediately centrifuged off, dissolved in dilute $NH_4OH$, purified by repeated precipitation, and finally dissolved in dilute $NH_4OH$.

Possible contaminating $P_i$ was removed from the RNA nucleotides by means of magnesia mixture with unlabelled $P_i$ added to it.

Portions of RNA and DNA fractions were used for radioactivity determinations and for reading the nucleic acid contents. The RNA content was determined by Mejbaum's (1939) orcinol reaction as modified by Ceriotti (1955), and the colour was read at 660 m$\mu$ on the Beckmann model DU spectrophotometer. The DNA content was determined by Dische's (1930) diphenylamine reaction as modified by Seibert (1940) and the colour was read at 600 m$\mu$ on the Beckmann model DU spectrophotometer. Specific activity was expressed as cpm/100 $\mu$g. of RNA-P and DNA-P, respectively.

*Relative specific activity.*—Following Smellie *et al.* (1955), the specific activity of the nucleotides and nucleic acids was referred to that of the $P_i$ in the blood of the experimental animal and stated as relative specific activity, *i.e.* :

$$\text{Relative specific activity} = \frac{\text{specific activity of fraction studied}}{\text{specific activity of blood } P_i} \times 10000$$

*Determination of specific activity of $P_i$ in the blood.*—The decapitated animal's blood was collected, and half its volume of trichloroacetic acid (20 per cent) was added to it. The mixture was centrifuged, and from the supernatant the $P_i$ was isolated in the cold with magnesium mixture (pH = 9) as $MgNH_4PO_4$. This was recrystallized, and dissolved in dilute acid. Portions of the solution were then used to determine $P_i$ by the method of Fiske and Subbarow (1925), as modified by Lohmann and Jendrassik (1926) and to measure radioactivity. Specific activity was expressed as cpm/100 $\mu$g. of $P_i$ ; as mentioned above, to this was referred the specific activity of the nucleotides and nucleic acids, respectively, to be stated as the relative specific activity.

*Isolation of RNA* for heterogeneity tests was performed by the method of Pain and Butler (1957). The specific activity of the RNA so obtained was expressed as cpm/100 $\mu$g. of ribose.

*Radiation count.*—Portion of the solution to be studied was measured into an aluminium dish and there allowed to dry. Radioactivity was then measured under an end-window GM tube, with the aid of an Orion 1871 type counter.

### RESULTS

Earlier animal experiments (Antoni *et al.*, 1958 ; Antoni, Vargha and Hidvégi, 1959) showed that for the isolation of RNA from liver labelled with $^{32}P$ *in vivo*, a 24-hour circulation time was the ideal. Even with subcellular particles this circulation time proved to be the best for labelling nucleic acids. Support for these findings has been provided by the work of Tyner, Heidelberger and LePage, (1953) on tumours, for which, in turn, we have established confirmation in respect of Guérin tumour.

TABLE I.—*Effect of Degranol on Relative Specific Activity of Tumour Nucleic Acids*

100 $\mu c$ of $^{32}P$/100 g. of body weight injected 24 hours before decapitation. The figures in brackets indicate the number of animals involved

| Duration of treatment | Relative specific activity | | |
| --- | --- | --- | --- |
| | DNA | nRNA | cRNA |
| Control | *3,950* (9)<br>3,530–4,200 | *10,650* (10)<br>9,150–11,020 | *8,300* (10)<br>6,950–8,750 |
| 24 hours | *1,850* (3)<br>1,410–2,460 | *7,530* (3)<br>7,100–9,820 | *8,050* (3)<br>7,050–8,250 |
| 48 hours | *1,520* (4)<br>1,120–1,860 | *6,810* (4)<br>6,170–7,830 | *7,250* (4)<br>6,810–7 750 |
| 72 hours<br>Experiment *a* | *920* (7)<br>720–1,040 | *4,950* (7)<br>4,610–6,050 | *4,220* (7)<br>3,790–4,540 |
| 72 hours<br>Experiment *b* | *2,900* (3)<br>1,940–3,720 | *10,920* (3)<br>9,100–11,430 | *9,210* (3)<br>7,110–9,850 |
| 96 hours | *4,420* (3)<br>3,840–4,960 | *11,250* (3)<br>10,620–12,700 | *8,820* (3)<br>8 070–9,440 |

Table I illustrates the effect Degranol exerted in our experiments on the incorporation of $^{32}P$ into tumoural nucleic acids. It shows that the drug caused their relative specific activity to decline at rates which followed a certain chronological regularity. The decrease was most marked in DNA : more than 50 per cent in 24 hours and reaching its minimum in 72 hours. It was less marked in nRNA and cRNA. At 96 hours after Degranol, *i.e.* at the time corresponding to the beginning of the restitutive stage as established by morphology, the relative specific activity of all the three nucleic acids studied was found to have returned to the level seen in the controls. In some cases, as early as 72 hours after treatment the relative specific activity was of the same order of magnitude in the experimental as in the control animals (cf. 72-hour experiment *b* in Table I). Histological examination showed the tumours of these animals to be in the restitutive stage.

Since in the current view the acid-soluble nucleotides are possible precursors of the nucleic acids our investigations were extended to the changes in their relative specific activity which follow treatment with Degranol. Table II illustrates the effect the drug had on the relative specific activity of acid-soluble nucleotides isolated simultaneously with nucleic acids and purified on active carbon. The data reveal that similar dynamic forces were at work in shaping the relative specific activity of both sources: the purified acid soluble nucleotides and the nucleic acids. In some of our experiments the number of counts, registered 72 hours after Degranol, was found closely to approach the limit of errors ; when averaging experimental results such counts were taken into consideration as non-radioactive values only.

TABLE II.—*Effect of Degranol on Relative Specific Activity of Acid-soluble Tumour Nucleotides*

100 $\mu$c of $^{32}$P/100 g. of body weight injected 24 hours before decapitation. The figures in brackets inicate the number of animals involved

| Duration of treatment | Relative specific activity |
|---|---|
| Control . . . | *310* (10)<br>262–340 |
| 24 hours . . . | *150* (3)<br>120–194 |
| 48 hours . . . | *82* (4)<br>47–113 |
| 72 hours . . .<br>Experiment *a* | *20* (7)<br>0–73 |
| 72 hours . . .<br>Experiment *b* | *194* (3)<br>153–260 |
| 96 hours . . . | *320* (3)<br>290–365 |

It was possible for us to verify our results in RNA fractions isolated by a different route (Pain and Butler, 1957).

The RNA was isolated from whole tumours, its specific activity was determined, and its heterogeneity studied (Antoni, Hidvégi and Lapis, 1960).

In Table III our experimental results are presented so as to demonstrate the *decrease* in the rate of $^{32}$P incorporation following treatment with Degranol ; in other words, the specific activity of the RNA in the tumour of the treated

TABLE III.—*Incorporation of $^{32}$P into RNA Isolated from Whole Tumour According to Pain and Butler* (1957)

100 $\mu$c of $^{32}$P/100 g. of body weight injected 24 hours before decapitation

| Duration of treatment | Specific activity $= \frac{\text{control cpm/100 } \mu\text{g. of ribose}}{\text{treated cpm/100 } \mu\text{g. of ribose}}$ |
|---|---|
| 24 hours . . . | *1·86* (3)<br>1·30–2·15 |
| 72 hours . . . | *2·77* (6)<br>2·27–4·16 |
| 96 hours . . . | *1·81* (3)<br>1·50–2·16 |

animal is referred to that in the tumour of the corresponding control animal. These data show that alterations induced by the drug in the rate of incorporation of $^{32}P$ follow the same trend in RNA isolated by a different method, namely, from whole tumour.

### DISCUSSION

Bodenstein (1947) was the first to study the effect of nitrogen mustard on nucleic acid metabolism. He found that the compound inhibited DNA synthesis, but assumed it had no inhibitory effect on the synthesis of RNA. (It would appear that the drug has no such effect on nuclei during mitosis, but does exert it in the interphase following mitosis.) Several authors hold similar views (Lowrance and Carter, 1950 ; Skipper *et al.*, 1951 ; Goldthwait, 1952). Harold and Zipporin recently found that mustard derivatives induced a transient inhibition of DNA synthesis in *Escherichia coli*, but allowed RNA and protein synthesis to continue unchanged.

Not unlike that of the other mustard derivatives, the initial effect of Degranol (between 6 and 24 hours) is to damage mitoses and bring down the total number of cell divisions. The peak effect falls between 48 and 72 hours, manifesting itself in extensive cellular and nuclear disintegration in the florid parts of the tumour, considerable expansion of necrotic areas, and the appearance of anomalous mitoses (cacomitoses) and multinuclear tumour changes are followed by the restitutive phase characterized by a cell population consisting predominantly of giant cells (Kellner and Németh, 1956 ; Kellner, 1956 ; Németh, Kellner and Lapis, 1958 ; Lapis and Németh, 1956*a*, 1956*b*).

On the evidence of our experimental results treatment with Degranol is followed by a decrease in the incorporation of $^{32}P$ into the nucleic acids and acid-soluble nucleotides of Guérin tumour. At the time it exerts its maximum effect the drug reduces the level of incorporation into DNA to about a quarter of the original. Incorporation into RNA and cRNA likewise shows decreases ; these however are less marked.

Our results, further, permit the statement that the changes in nucleic acid metabolism seen after Degranol are a dynamic process, the same as the morphological changes. In 24 hours a single dose decreases the relative specific activity of DNA by 50 per cent and reduces the total number of cell divisions. The drug exerts its maximum effect between the 48th and 72nd hour after input. This is the time when the morphological picture shows extensive cellular and nuclear disintegration and a progressive decline to the minimum of the relative specific activity of the nucleic acid fractions. This gradual decrease is particularly marked in DNA, a phenomenon which fairly corresponds to the extensive nuclear disintegration revealed by morphology. Conformity with the morphological picture is still more pronouned at 96 hours, when the relative specific activity of DNA, nRNA, and cRNA is observed to have returned to the level seen in the controls, coinciding with the appearance of multinuclear giant cells and amitoses in numbers so great as to be practically the only building stones of the entire florid tumours.

Conflicting interpretations of these cell forms are offered in the literature. Many authors have brought them into connection with regressive processes. The view, however, is gaining ever more adherents that they are very frequent and interesting forms of cell division. Kellner's studies (1952, 1960) also favour the idea that these kinds of cells have a decisive part to play in the restitutive processes that follow damages caused by chemotherapeutics. By our findings, at the time these cells come into prominence the level of relative specific activity of DNA rises to that observed in the controls, which phenomenon seems to confirm that their appearance may actually be connected with processes progressive, and not regressive.

While in some cases, at 72 hours after Degranol the relative specific activity was the same in the experimental animals as in the controls, in others it showed the low level which characterized the maximal effect of the drug. Such differences, however, were invariably found to conform with the changes established by morphology. For, whenever the relative specific activity was as high as in the controls, the histological picture revealed changes typical of the restitutive stage ; conversely, whenever it was low, histology still showed marked nuclear destruction.

It seems safe to claim that our results offer the possibility of quantitatively characterizing the alterations and their individual phases, to which the mustard derivatives give rise in the tumour—and which hitherto could only be differentiated qualitatively on the strength of morphological changes—by measuring the incorporation of $^{32}P$ into the nucleic acids.

SUMMARY

The compound 1, 6-bis-($\beta$-chloroethylamino)-1, 6-dideoxy-D-mannitol-dihydrochloride (Degranol ; BCM) has been studied for its effects on the nucleic acid metabolism of Guérin carcinoma transplanted into rats. A single dose of the drug was found to decrease incorporation of $^{32}P$ into the various nucleic acid fractions. At 72 hours after injection the decrease was most marked in DNA. It was less marked in nRNA and cRNA. At 96 hours, $^{32}P$ incorporation into all the three nucleic acids studied was seen to have returned to the levels observed in the control animals. Variations of a similar trend were noted in the acid-soluble nucleotide fractions isolated simultaneously with the nucleic acids.

The conclusion is drawn that the changes occurring in the nucleic metabolism of tumours represent a dynamic process, as do the morphological changes.

### REFERENCES

ANTONI, F., HIDVÉGI, E. J. AND LAPIS, K.—(1960) *Nature, Lond.*, **186**, 81.
*Idem*, JOSEPOVITS, G., HIDVÉGI, E. J. AND SZÉKESSY-HERMANN, V.—(1958) *4th Int. Congr. Biochem.*, Vienna.
*Idem*, VARGHA, L. AND HIDVÉGI, E. J.—(1959) *Acta physiol. Acad. Sci. hung.*, **16**, 1.
BODENSTEIN, D.—(1947) *J. exp. Zool.*, **104**, 311.
CERIOTTI, G.—(1955) *J. biol. Chem.*, **214**, 59.
DAVIDSON, J. N. AND SMELLIE, R. M. S.—(1952) *Biochem. J.*, **52**, 594.
DISCHE, Z.—(1930) *Microchemie*, **8**, 4.
FISKE, C. H. AND SUBBAROW, Y.—(1925) *J. biol. Chem.*, **66**, 375.
GOLDTHWAIT, D. A.—(1952) *Proc. Soc. exp. Biol. N.Y.*, **80**, 503.
HEALY, G. M., SIMINOVITCH, L., PARKER, R. C. AND GRAHAM, A. F.—(1956) *Biochim. Biophys. Acta*, **20**, 425.
HECHT, L. I. AND POTTER, V. R.—(1956) *Cancer Res.*, **16**, 988.
HERSHEY, A. D.—(1954) *J. gen. Physiol.*, **38**, 145.
HOGEBOOM, G. H., SCHNEIDER, W. C. AND STRIEBICH, M. J.—(1952) *J. biol. Chem.*, **196**, 111.
HURLBERT, R. B. AND POTTER, V. R.—(1952) *Ibid.*, **195**, 257.

KELLNER, B.—(1952) *MTA Biol. és Orv. Tud. Osztályának Közleményei* (*Transactions of the Biological and Medical Section of the Hungarian Academy of Sciences*—Hungarian only), **7**, 435.—(1960) *Acta Un. int. Cancr.*, **16**, 80.—(1956) *Acta morph. hung.*, **7**, 215.

*Idem* AND NÉMETH, L.—(1956) *Z. Krebsforsch.*, **61**, 165.

LAPIS, K. AND NÉMETH, L.—(1956*a*) *Klin. Wschr.*, **34**, 864.—(1956*b*) *Brit. J. Cancer*, **10**, 719.

LOHMAN, K. AND JENDRASSIK, L.—(1926) *Biochem. Z.*, **178**, 419.

LOWRANCE, P. B. AND CARTER, C. E.—(1950) *J. cell comp. Physiol.*, **35**, 387.

MEJBAUM, W.—(1939) *Z. physiol. Chem.*, **258**, 117.

NÉMETH, L., KELLNER, B. AND LAPIS, K.—(1958) *Ann. N.Y. Acad. Sci.*, **68**, 879.

NYGAARD, O. AND RUSCH, H. P.—(1955) *Cancer Res.*, **15**, 240.

PAIN, R. H. AND BUTLER, J. A. V.—(1957) *Biochem. J.*, **66**, 299.

PILERI, A. AND LEDOUX, L.—(1957) *Biochim. Biophys. Acta*, **26**, 309.

SEIBERT, F. B.—(1940) *J. biol. Chem.*, **133**, 593.

SHIVE, W. AND SKINNER, C. G.—(1958) *Annu. Rev. Biochem.*, **27**, 643.

SKIPPER, H. E. AND BENNETT, L. L. Jr.—(1958) *Ibid.*, **27**, 137.

*Idem*, MITCHELL, J. H. Jr., BENNETT, L. L. Jr., NEWTON, M. A., SIMPSON, L. and EIDSON, M.—(1951) *Cancer Res.*, **11**, 145.

SMELLIE, R. M. S., HUMPHREY, G. F., KAY, E. R. M. AND DAVIDSON, J. N.—(1955) *Biochem. J.*, **60**, 177.

TYNER, E. P., HEIDELBERGER, C. AND LEPAGE, G. A.—(1953) *Cancer Res.*, **13**, 186.

VARGHA, L.—(1955) *Naturwissenschaften*, **42**, 582.